# How self-awareness is connected to less experience of action crises in personal goal pursuit

**DOI:** 10.1007/s11031-022-09942-5

**Published:** 2022-05-23

**Authors:** Antonia Kreibich, Benjamin Mario Wolf, Martin Bettschart, Mirjam Ghassemi, Marcel Herrmann, Veronika Brandstätter

**Affiliations:** 1 Department of Psychology, University of Zurich, Binzmuehlestrasse 14/6, 8050 Zurich, Switzerland; 2 School of Applied Psychology, Pfingstweidstrasse 96, 8005 Zurich, Switzerland

**Keywords:** Action crisis, Self-awareness, Problem-solving orientation, Goal pursuit, Individual differences

## Abstract

In this research, we applied a differential perspective to the study of *action crises*, i.e., being in an intra-psychic decisional conflict whether to pursue or abandon a goal once difficulties in its pursuit arise. In two studies, we investigated the role of individuals’ levels of *self-awareness* when experiencing such action crises. Both among professional ballet dancers (daily diary, Study 1) and university undergraduates (preregistered experience sampling, Study 2), individuals with greater levels of (dispositional and situational) self-awareness showed an adaptive, that is, *problem-solving* oriented way of dealing with difficulties in the pursuit of their (training or study) goals. As a consequence, self-awareness contributed to less experience of action crisis during goal pursuit and, as a result, led to better goal performance.

**Supplementary Information:**

The online version contains supplementary material available at 10.1007/s11031-022-09942-5.

## Introduction

Life is a continual process of setting goals and acting to achieve them (Carver & Scheier, 1998). However, goal pursuit does not always run smoothly. If difficulties such as obstacles or setbacks repeatedly occur on the way to goal attainment, one might experience a decisional conflict between the further pursuit of or disengagement from one’s personal goal, that is, an *action crisis* (Brandstätter & Schüler, 2013). This critical phase in goal striving has detrimental consequences for well-being and goal-related performance (e.g., Brandstätter et al., 2013; Herrmann & Brandstätter, 2015; Venhorst et al., 2018).

Given these negative consequences of experiencing an action crisis, it is important to identify possible antecedents. We start off with empirical evidence on the predictive power of goal-related difficulties for experiencing an action crisis (Bettschart et al., 2019; Ghassemi et al., 2017, 2020). Specifically, we asked ourselves: *Who* is better in dealing with difficulties and thereby preventing action crises? And *how* exactly do they do so?

### The experience of action crises in goal pursuit

An action crisis is defined as a decisional conflict between the further pursuit of and disengagement from a goal after the individual has already invested considerable time or other resources and prospects have worsened (Brandstätter & Schüler, 2013). In the course of an action crisis, the individual re-evaluates his or her goal. Yet, it is often anything but clear what the best option would be—to hang on or to let go—and so action crises set the stage (Herrmann & Brandstätter, 2015) for disengagement but do not inevitably result in it.

An action crisis transforms how an individual is attuned to their goals (Herrmann et al., 2014), thus the concept encompasses further phenomena beyond decisional conflict, such as procrastination, disorientation, diminishing progress (Brandstätter & Schüler, 2013) and especially goal-related doubts (Ghassemi et al., 2020). Such a phase of indecision and resulting stagnation in a personally relevant goal is stressful and therefore impairs well-being (e.g., Brandstätter et al., 2013), physical health (Wolf et al., 2019), and goal-related performance (athletic context, e.g., Brandstätter et al., 2013; Venhorst et al., 2018; academic context, e.g., Herrmann & Brandstätter, 2015).

Research on the antecedents of action crises has shown the strong predictive power of experiencing difficulties in goal pursuit and a low appraisal of the goal’s attainability (Bettschart et al., 2019; Ghassemi et al., 2017, 2020). Setting autonomous goals (i.e., out of personal interest rather than external rewards) that are typically related to fewer difficulties is therefore associated with less action crisis (Holding et al., 2017). This adaptive way of goal setting is the case for individuals with higher levels of mindfulness (Marion-Jetten et al., 2021) and self-efficacy (Wolf et al., 2018), and stronger emotion regulation capacities (action orientation; Herrmann & Brandstätter, 2013). Besides adaptive goal setting, the dealing with difficulties is of importance. Specifically, individuals with strong emotion regulation capacities (i.e., action-oriented individuals) can overcome the negative emotions that result from difficulties in goal pursuit more readily and thus do not easily run into action crises (Herrmann & Brandstätter, 2013; Wolf et al., 2018). In addition to this *affective* reaction, the individual’s *cognitive* reaction to difficulties in goal pursuit is important as well (Kreibich et al., 2021), but to date not investigated in the realm of action crises research. Adopting a so-called *problem-solving orientation* when being confronted with difficulties, that is, an orientation towards the search for and selection of appropriate strategies and instrumental means, has been shown to be highly helpful in solving these difficulties (e.g., Lazarus & Folkman, 1984) and therefore in maintaining the goal’s attainability. Thus, it therefore seems reasonable to assume that individuals who face goal-oriented difficulties in a problem-solving oriented way are better able to overcome them and are less likely to experience action crises. This is *how* these individuals might do so, but *who* are they? Previous findings (Kreibich et al., 2021) suggest that such an orientation is more likely for individuals with stronger *self-awareness.*

### The role of self-awareness for self-regulation

Self-awareness refers to a metacognitive state in which the individual pays attention to aspects of the self, that is, their thoughts, feelings, and behavior (i.e., Duval & Wicklund, 1972). While early research has investigated momentary states of self-awareness (objective self-awareness, Duval & Wicklund, 1972), later research has focused on individual differences in self-awareness across situations (private vs. public self-consciousness, Fenigstein et al., 1975; meta-cognitive self-reflection vs. insight, Grant et al., 2002; rumination vs. reflection, Trapnell & Campbell, 1999). For the sake of simplicity, we use in this research the term *situational self-awareness* to refer to intraindividual differences and *dispositional self-awareness* to refer to interindividual differences in self-awareness.

Most of the above mentioned conceptualizations of self-awareness indicate the important role that being in a (situational or dispositional) state of self-awareness plays in self-regulation (Duval & Wicklund, 1972; Fenigstein, 2009; Grant et al., 2002). Self-awareness leads individuals to focus on their goals, which unleashes self-regulatory processes that support goal attainment (Baumeister et al., 1994; Carver & Scheier, 1998). More concretely, individuals in states of heightened self-awareness are thought to monitor their goal pursuit by comparing their current states in goal pursuit with their goals, identify any discrepancies between them, and reduce them through discrepancy-reducing, and thus goal-related, behavior (Carver & Scheier, 1998).

This goal monitoring process distinguishes self-awareness as we conceptualize it from other notions of self-awareness. For instance, *rumination* describes maladaptive, frequently recurring thoughts that are motivated by anxiety about the self (Trapnell & Campbell, 1999). Although self-awareness and rumination are empirically linked with each other (e.g., Silvia & Phillips, 2011), they differ in certain points. First, ruminative thoughts are not necessarily self-relevant (Martin & Tesser, 1996), whereas goal monitoring oriented thoughts in a state of self-awareness indeed are self-relevant (Duval & Wicklund, 1972). Second, compared to rumination, self-awareness in its original sense is defined regardless of mood and motives (Fenigstein et al., 1975). Lastly, their differences become apparent with regard to their relations with personality dimensions. While rumination is connected to neuroticism, self-awareness shows associations with openness to experience and conscientiousness (Panah & Seif, 2014). Furthermore, self-awareness is distinct from *mindfulness*, another form of self-directed attention that has received growing interest in psychological literature (Brown & Ryan, 2003; Davidson & Dahl, 2018). Mindfulness describes a state of being aware of the present moment and showing an accepting attitude towards this moment (Bishop et al., 2004). Thus, while self-awareness is characterized by goal monitoring and evaluating goal-related states, mindfulness is a form of non-evaluative self-directed attention (e.g., Evans et al., 2009).

Most importantly to this research, self-awareness supports goal pursuit particularly in situations when difficulties arise in goal pursuit (Burwell & Shirk, 2007; Jäkel & Schreiber, 2013). In such situations, self-awareness’ distinctive process of goal monitoring detects discrepancies between the current state in goal pursuit (i.e., obstacles that jeopardize smooth goal progress) (e.g., Oettingen & Gollwitzer, 2002) and the goal. Thus, in order to reduce this goal discrepancy, means that are highly instrumental in overcoming these difficulties must be searched for and selected. It has been shown that self-aware individuals automatically adopt such a so-called problem-solving orientation when being confronted with goal-related difficulties (Kreibich et al., 2021). Such a problem-solving orientation is therefore an adaptive approach for dealing with difficulties as opposed to more maladaptive approaches, such as negative palliation or avoidance (Bodenmann, 2000; Lazarus & Folkman, 1984).

By searching for and selecting instrumental means of dealing with difficulties, self-aware individuals can take action to make further progress toward their goal (Kreibich et al., 2021). Their problem-solving-oriented way of handling goal-related difficulties therefore helps self-aware individuals to persist in the face of difficulties and to maintain the attainability of the goal and in turn might therefore reduce the experience of action crises (Bettschart et al., 2019; Brandstätter & Schüler, 2013; Ghassemi et al., 2017).

### The present research

In our research, we therefore hypothesized (and preregistered) that self-awareness is positively associated with a problem-solving orientation in goal pursuit and, in turn, connected to less experience of action crises. We investigated this hypothesis for the training goals of professional ballet dancers (Study 1) and the academic goals of university undergraduates (Study 2)—two goal domains that pose considerable challenges. Moreover, we examined both between-person effects, by investigating dispositional self-awareness (Study 1, 2), and within-person effects, by studying situational levels of self-awareness (Study 2). Furthermore, we explored the consequences of this hypothesized mediation effect on (subjectively assessed) daily goal progress and (objectively assessed) long-term goal performance (Study 2).

### Statistical analyses

Correlations and hierarchical multiple regression analyses were computed using R (R Core Team, 2014). For simple mediation analyses, we used the “mediation” package for R (Tingley et al., 2014). Mediation analyses with multilevel data and several sequential mediators were conducted using the “sermedML” and “sermedMLM” scripts (Wolf, 2020). In line with recommendations regarding Open Science (Simmons et al., 2012), we are reporting all excluded data, how we determined our sample size, and all measures. Importantly, Study 2 was preregistered (see our time-stamped preregistration: https://aspredicted.org/r33qp.pdf).^1^ Furthermore, all study materials (i.e., methods, detailed description of study design) of both studies are publicly available on the Open Science Framework (https://osf.io/kcx7f/). The data set and analysis code for Study 1 is also available online on OSF. The data sets and analysis codes for Study 2 are available upon request from the last-named author. The study procedure conformed to the requirements of the local research ethics board and the Ethics Code of the American Psychological Association.

## Study 1

To replicate prior research on the relation between dispositional self-awareness and problem-solving orientation (Kreibich et al., 2021), and to gather first empirical evidence on how their relationship affects the experiencing of action crises, we assessed these variables as part of a larger daily diary study.^2^ In this study, dancers had to report about a specific goal, namely the goal of making their training as effective as possible (referred to below as their “training goal”).

### Method

#### Participants

For this study, we recruited as many professional ballet dancers as possible in a given period of four weeks. We recruited participants world-wide via mailing lists, bulletin boards, and social media. In return for participating in the study, participants received a 15 Euro Amazon voucher, access to an online webinar on psychological phenomena in dance, and the chance to win an additional 200 Euro Amazon voucher. In this study, 80 ballet dancers (86% female) with a mean age of 23.6 (*SD* = 5.4) took part, coming from 25 different countries (69% Central Europe, 13% Eastern Europe, 11% Western Europe, 4% Asia, 2% South America, 1% Australia). The dancers were either employed in a company or theatre (*n* = 24), or in the free dancing scene (*n* = 22), or engaged in other dance (education) projects (*n* = 34).

#### Procedure

The study was designed as an online diary study, but only data from the baseline questionnaires one week prior to the daily measurements was used for the investigation of the hypothesis presented here. The baseline questionnaire for this study comprised an informed consent form, sociodemographic variables and, among others, measurements to assess dancers’ dispositional levels of self-awareness, their problem-solving orientation in goal pursuit and their levels of experiencing an action crisis with respect to their training goal.

##### Self-awareness

Dispositional self-awareness was assessed using the 12-item Self-Reflection subscale of the Self-Reflection and Insight Scale (SR-SRIS; Grant et al., 2002). This scale assesses one’s awareness of inner thoughts (e.g., “I frequently take time to reflect on my thoughts.”), inner feelings (e.g., “I frequently examine my feelings.”), and behavior (e.g., “It is important for me to evaluate the things that I do.”). The response scale ranged from 1 (*strongly disagree*) to 6 (*strongly agree*) (*M* = 5.12, *SD* = 0.81, α = 0.89).

##### Problem-solving orientation

In order to assess participants’ orientation towards problem-solving in goal pursuit, we used the Individual Coping Questionnaire (INCOPE; Bodenmann, 2000). In this questionnaire, the dancers were asked to indicate how they had dealt with difficulties regarding their training goals within the last two weeks. Using 21 items, the INCOPE assesses a variety of both functional (e.g., problem-solving) and dysfunctional (e.g., evasion) forms of coping. The coping strategy problem-solving was originally assessed using six items, but we used two of them:^3^ “I think very carefully about what’s going on and what to do” and “I actively influence the situation” (*r* = 0.39, *p* < 0.001). Scales ranged from 1 (*never*) to 5 (*often*) (*M* = 3.53, *SD* = 0.85).

##### Action crisis

The dancers’ levels of action crisis with respect to their training goals were assessed using the 6-item Action Crisis Scale (ACRISS; Brandstätter & Schüler, 2013). This scale assesses different aspects of an action crisis which we adapted to the training context, e.g. goal-related doubts (“I sometimes doubt whether I should continue my training or disengage from it.”) or a disengagement impulse (“I have thought several times of disengaging from my training.”). The response scale ranged from 1 (*strongly disagree*) to 7 (*strongly agree*) (*M* = 3.45, *SD* = 1.22, α = 0.75).

### Results

**Fig. 1:**
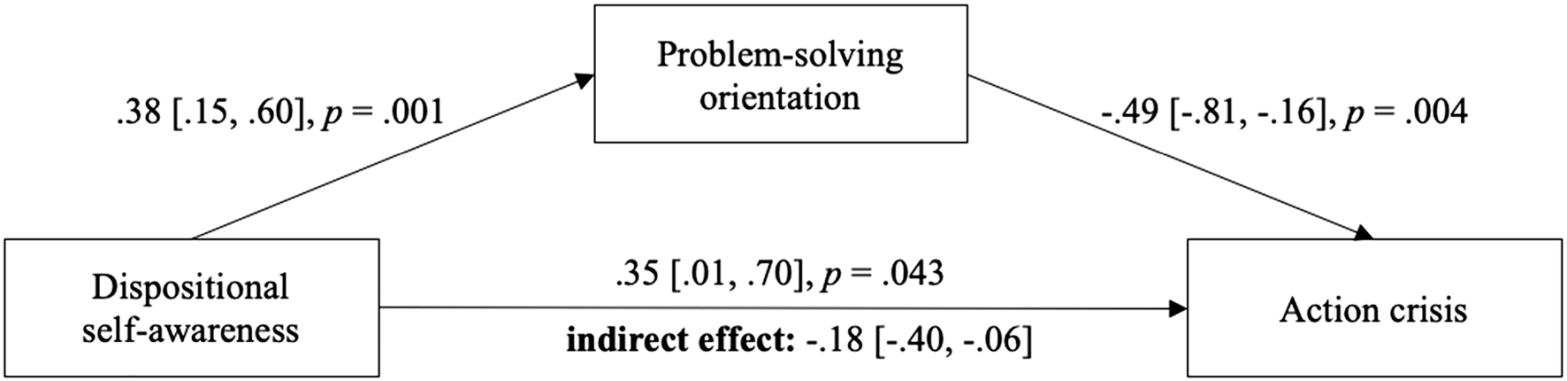
Mediation model from Study 1 (*N* = 80). The 95% bias-corrected confidence intervals of the unstandardized coefficients were calculated using bootstrapping (5000 samples). The *p*-value for the indirect effect is also based on bootstrapping

In order to test our hypothesis, we conducted mediation analyses investigating the effect of dispositional self-awareness on the experience of action crises mediated by problem-solving orientation. The results of the mediation analyses are presented in Fig. 1. As expected, dancers with greater levels of dispositional self-awareness reported a greater problem-solving orientation in dealing with difficulties in their training (path *a*). Moreover, a problem-solving orientation was connected to less experience of action crises with respect to their training goals (path *b*). Thus, we found the expected indirect effect indicating that self-aware dancers reported less experience of action crises in line with their distinctive way of dealing with goal-related difficulties, namely problem-solving oriented (path *c’*).^4^ However, we also found an unexpected positive direct effect of self-awareness on action crisis.

### Discussion

Study 1 provides some first empirical evidence for the hypothesized mediation effect among professional ballet dancers. As expected, ballet dancers with greater levels of dispositional self-awareness reported a greater problem-solving orientation in the pursuit of their training goals. This, in turn, reduced the experience of action crises. Unexpected was the positive direct association between dispositional self-awareness and action crisis. To figure out, whether this finding was due to the very specific sample or not, we conducted another study. The aim was also to replicate the effect in a larger and less specific sample and furthermore to not only investigate between-person but also within-person variations. We furthermore were interested in investigating whether this proposed mediation effect had practical relevance by investigating its consequences for actual goal performance. Hence, we conducted an experience sampling study with university undergraduates.

## Study 2

This study focuses on the goal of undergraduate students to complete a bachelor’s degree (referred to below as their “academic goal”) and tracked their goal-related experiences over the course of two weeks using the experience sampling method.^5^ This allows us to disaggregate between-persons and within-person effects in the variables of interest (Curran & Bauer, 2010). Please note that we are here only reporting the central analysis of our hypothesis; for more detailed results, please consult the supplementary online materials.

### Method

#### Participants

We recruited undergraduate students using flyers, notices in lectures and mailing lists. We intended to recruit close to 250 participants, as correlations tend to stabilize at this sample size (Schönbrodt & Perugini, 2018). We were able to recruit 262 participants (69% female, aged 18–40) with a mean age of 21.9 (*SD* = 2.6). Due to dropouts before and during experience sampling, the effective sample size for the between-person analyses is 215 (70% female; aged 18–40, *M* = 22.0, *SD* = 2.54). Full participation was compensated with 50 Swiss francs or partial course credits. Response rates of 90% or higher in the experience sampling phase were requited with 20 Swiss francs. More details about data collection and participant dropout are given in the supplemental materials.

##### Procedure

The study involved a baseline questionnaire (T1), a two-week experience sampling phase, and a follow-up questionnaire (T2). After registering, participants answered the baseline questionnaire, which included study information, a consent form, dispositional measures, and demographic questions. In the subsequent experience sampling phase, participants received brief questionnaires on their mobile phones at five randomized times of each working day. One week after the end of the experience sampling period, participants responded a short follow-up questionnaire to assess action crises’ experience again.

#### Measures

The baseline questionnaire included the SR-SRIS (Grant et al., 2002) to measure *dispositional self-awareness* (*M* = 4.57, *SD* = 0.77, α = 0.89), the ACRISS (Brandstätter & Schüler, 2013) for *action crisis* regarding the academic goal (*M* = 2.99, *SD* = 1.06, α = 0.79), and the same two items from the INCOPE (Bodenmann, 2000) as in Study 1 to measure *problem-solving orientation* (*M* = 3.66, *SD* = 0.69, *r* = 0.35, *p* < 0.001). The ACRISS was also included in the follow-up questionnaire (*M* = 2.92, *SD* = 0.96, α = 0.75).

#### Baseline and follow-up questionnaire

#### Experience sampling phase

The last of the five brief daily questionnaires administered did not include all the relevant measures and was not used for the present analyses. In the four questionnaires per day that were used, all items focused on the activity participants had primarily been engaged in during the past hour. For the present analyses, we only used responses for which participants categorized their previous activity as being *study-related*. An average of 14 relevant questionnaires (range: 1–31, *SD* = 5.45) was available per participant. Unless otherwise specified, all items were answered on a scale from 1 (*strongly disagree*) to 7 (*strongly agree*)*.*

To assess *situational self-awareness*, participants evaluated one statement adapted from the SRIS-SR (Grant et al., 2002): “I have thought about myself (my thoughts, my feelings, my behavior)” on a scale from 1 (*never*) to 7 (*very frequently*) (*M* = 3.07, *SD* = 1.96).

To measure *momentary problem-solving orientation*, participants indicated their agreement with one statement adapted from the INCOPE (Bodenmann, 2000): “I have been thinking very carefully about what to do and how to do it” (*M* = 3.98, *SD* = 1.67).

For a state indicator of an ongoing *action crisis*, we focused on doubts about further goal pursuit. Because smooth goal pursuit is associated with a focus on the positive aspects of the goal and the necessary steps to attain it (Heckhausen & Gollwitzer, 1987), goal-related doubts are a clear cognitive marker of action crisis experience. In another analysis of Study 2, the person mean of doubts was strongly correlated with the full action crisis scale (*r* = 0.57—0.67, *p* < 0.001, Ghassemi et al., 2020). It can therefore be used to measure experience of an action crisis at a specific moment and within very limited survey time.

We used three items to measure action crisis, two of which are adapted from the ACRISS (Brandstätter & Schüler, 2013): “I have doubts about whether I should continue or quit my studies”, “I am certain that pursuing my studies is the right thing to do for me” (inverted, not ACRISS but new), “I am thinking about giving up my studies” (*M* = 1.78, *SD* = 1.07, α = 0.85).

Subjective *goal progress* was also measured using one item referring to study-related activities prior to answering the brief questionnaire: “I made good progress” (*M* = 4.65, *SD* = 1.42).

#### Performance indicator

In order to test a more objective indicator of progress towards the academic goal, we later obtained the academic record of the students for the relevant semester from the university administration. These records included grades and ECTS points^6^ awarded at the end of the semester. All participants agreed to let us use this data for anonymized research purposes. We calculated the average course grade weighted by the courses’ ECTS points as a semester grade point average (GPA). Ungraded courses were not used and, because some students had no graded courses in that semester, the sample size is reduced to *N* = 189 for analyses involving the GPA. The mean GPA was 4.72 (*SD* = 0.88)^7^ and the mean ECTS points collected were 23.99 (*SD* = 7.29).

### Results

**Fig. 2:**
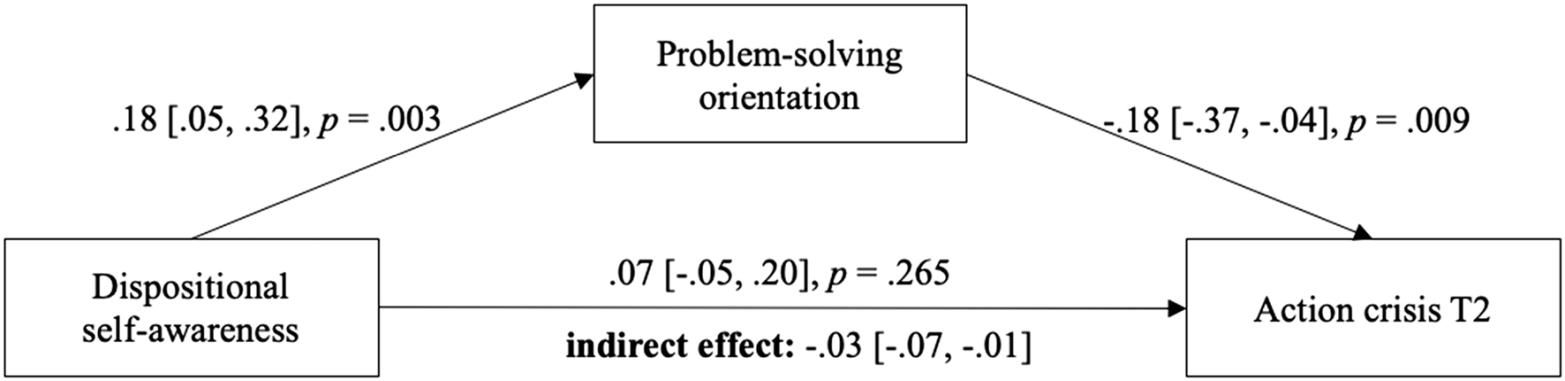
Mediation model from Study 2 (*N* = 215). Analyses are controlled for action crisis at T1. The 95% bias-corrected confidence intervals of the unstandardized coefficients were calculated using bootstrapping (5000 samples). The *p*-value for the indirect effect is also based on bootstrapping

We first tested our hypothesis on the between-person level using data from the baseline- (T1) and follow-up (T2) questionnaires. Specifically, we assessed the indirect effect of dispositional self-awareness (T1) via problem-solving orientation (T1) on action crisis experience (T2). To avoid confounding by a pre-existing action crisis experience, we included baseline action crisis (T1) as a covariate. As expected, the two measures of action crisis (T1 and T2) were strongly associated, *b* = -0.62, CI 95% = [0.45, 0.72]. The results of the mediation analysis are given in Fig. 2. As in Study 1, the indirect effect of dispositional self-awareness via problem-solving orientation was negative and statistically significant. Contrary to Study 1, the direct positive effect of dispositional self-awareness on action crisis was not significant. Consequently, the total association of dispositional self-awareness and action crisis was not significant, too (*r* = 0.08, *p* > 0.05, see Suppl. Table 4). When removing baseline action crisis (T1) from the model, we again find a statistically significant positive direct effect of dispositional self-awareness on action crisis (T2; *b* = 0.17, CI 95% = [0.01, 0.33], see Suppl. Table 6).

**Fig. 3:**
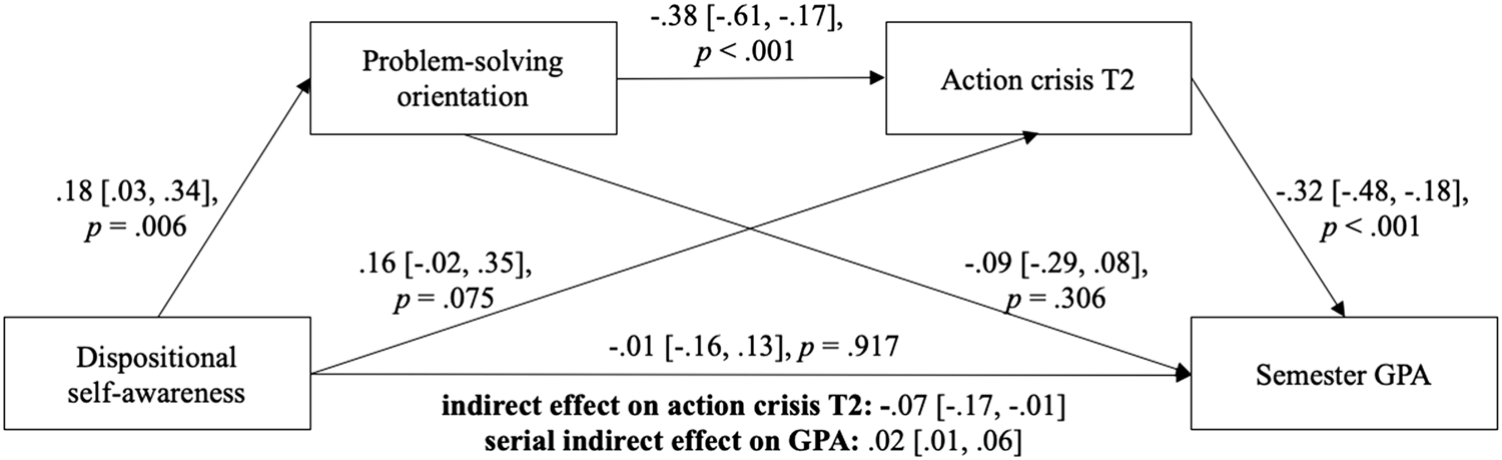
Serial mediation model with multiple mediators from Study 2. The 95% bias-corrected confidence intervals of the unstandardized coefficients were calculated using bootstrapping (5000 samples). *N* = 189

As a further exploration, we tested whether this mediation effect accounted for better goal-related performance by conducting a serial mediation analysis of students’ semester GPA as an objective indicator of goal performance. This model is *not* an extension of the first mediation model (Fig. 2) because the sample was reduced for GPA. As Fig. 3 illustrates, dispositional self-awareness (T1) had a positive serial indirect effect on GPA via problem-solving (T1) and action crisis (T2), and this effect was statistically significant. Again, there was no significant direct relationship between self-awareness and action crisis.

**Fig. 4:**
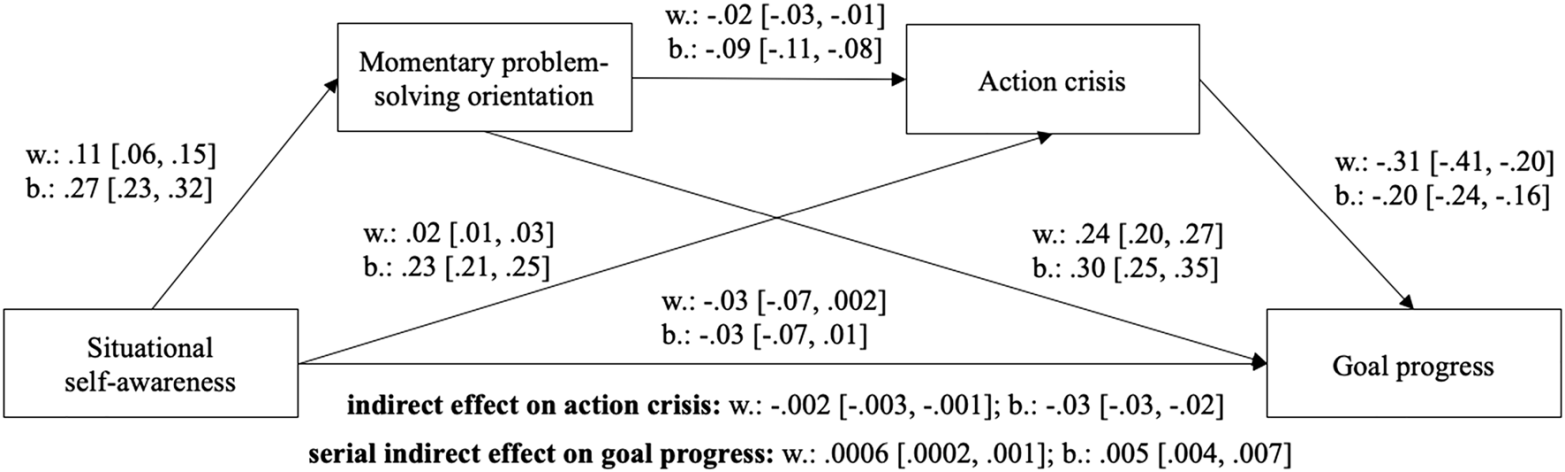
Mediation model with multiple mediators from Study 2. The 95% bias-corrected confidence intervals of the unstandardized coefficients were calculated using bootstrapping (5000 samples). w.: within-person effect, b.: between-person effect. *N* = 3512 questionnaires from 250 participants. *p*-values for each path are reported in the supplemental material

Furthermore, we tested the same hypothesis using data from the experience sampling phase and dissociated within- vs. between-person effects. Following recommendations for intensive longitudinal data (Bolger & Laurenceau, 2013), the predictor (incl. mediator) variables were each split into a person means variable and a person-centered variable. Thus, for each regression path, we obtained a within- and a between-persons effect. All the direct effects of this model are shown in Fig. 4. The indirect effect of self-awareness on action crisis via problem-solving was negative and statistically significant both for the within-persons and between-persons level, supporting our hypothesis. Interestingly, in the analyses with the daily data, the positive correlation between self-awareness and action crisis that we found in Study 1 was again significant, both on between-person and within-person levels. Thus, the total association of self-awareness and action crisis on the state level was positive (*r* = 0.12, *p* < 0.01, see Suppl. Table 4).

Figure 4 also depicts our explorative extension of the mediation model by investigating its effect on daily goal-related performance. Specifically, we tested whether self-awareness has a negative serial mediation effect, via problem-solving and action crisis, on goal progress as a subjective indicator of goal performance. As Fig. 4 indicates, the serial indirect effect of self-awareness on goal progress via state problem-solving orientation and action crisis was negative and statistically significant on both data levels.

### Discussion

To sum up the results of Study 2, we were able to replicate our findings from Study 1 showing the negative effect of dispositional self-awareness on action crisis via problem-solving. We furthermore found evidence of the hypothesized negative association when analyzing situational states of self-awareness, problem-solving and action crisis. The indirect negative effect was found both for between-person differences and when analyzing within-person fluctuations in self-awareness states. Furthermore, we found initial evidence that high problem-solving orientation and a low level of action crisis form a pathway through which self-awareness indirectly promotes students’ goal performance—both on a daily base by showing that self-aware individuals report a greater daily goal progress, and on the long run by showing self-aware students’ better grades in their exam. Interestingly, in some of our analyses we found again the positive association between self-awareness and action crises. Hence, this effect is not only due to the specificity of the sample in Study 1 and deserves closer consideration below.

## General discussion

In this research, we investigated whether self-aware individuals experience less goal-related action crises due to their greater problem-solving orientation in dealing with goal-related difficulties. Indeed, we found first empirical evidence for this mediation effect among professional ballet dancers pursuing training goals (Study 1). We successfully replicated this finding—on both between- and within-person levels—in an experience sampling study in a sample of university undergraduates pursuing academic goals (Study 2). Moreover, we found that the hypothesized mediation effect predicts better goal-related performance both subjectively assessed using participants’ self-reported goal progress and objectively assessed using participants’ exam grades.

### Theoretical implications

With this research, we are contributing to the study of action crises, as a critical instance in goal-directed self-regulation, in several ways. First of all, we contribute to increasing research on *antecedents* of action crises that constitute a sensitive phase in goal pursuit with severe consequences for health, well-being, and performance (e.g., Brandstätter et al., 2013; Wolf et al., 2018, 2019). An antecedent of experiencing action crises is the occurrence of severe difficulties in goal pursuit (Bettschart et al., 2019). Given that person-related antecedents play a major role in explaining differences in self-regulation (Hoyle, 2006), prior action crisis research has focused on individual differences in preventing difficulties via adaptive goal setting (e.g., Holding et al., 2017). Moreover, research has focused on the benefits of emotion regulation capacities for dealing affectively with difficulties in goal pursuit (Herrmann & Brandstätter, 2013). The research presented here takes a different approach to dealing with difficulties. More concretely, we widen the *cognitive perspective* on antecedents of action crises by investigating the role of the problem-solving orientation. We are certainly not the first to highlight the adaptive role of problem-solving in goal pursuit (Bodenmann, 2000; Lazarus & Folkman, 1984). However, our research highlights the idea that viewing difficulties as problems and searching for solutions in order to solve these problems can prevent action crises. Moreover, we suggest that a problem-solving orientation might be especially helpful in preventing action crises as it enables individuals to search for and select instrumental means of overcoming the difficulties (Kreibich et al., 2021). As a result, individuals can perform instrumental actions to make further progress toward their goals. Thus, a problem-solving orientation might be especially helpful because it helps individuals to maintain the attainability of their goal, and this contributes to earlier research proving that low goal attainability is a strong predictor of action crises (Ghassemi et al., 2017). Eventually, we replicate the detrimental effect of action crises on an objective measure of academic performance (Herrmann & Brandstätter, 2015), and even more important, show how individual differences contribute to this relationship.

Furthermore, we not only investigated *how* to deal with difficulties in the best possible way, but also identified *who* is most likely to do so and therefore less prone to experiencing action crises. More concretely, we investigated individual differences in *self-awareness* (Grant et al., 2002). Much research has addressed maladaptive forms of self-directed attention, namely rumination (Nolen-Hoeksema et al., 2008; Trapnell & Campbell, 1999), or non-evaluative ones like mindfulness (Bishop et al., 2004). Both concepts are also investigated in the realm of action crisis research showing that goal-related doubts coming along with an action crisis resemble ruminative thinking (Brandstätter et al., 2013) and showing that mindful individuals are protected from experiencing action crises because they set autonomous goals that typically come with fewer goal difficulties (Marion-Jetten et al., 2021). However, it is well conceivable that even the best goal selection does not help to prevent all difficulties. Thus, it is crucial to find an adaptive way to deal with these difficulties. Self-awareness plays a key role in self-regulation because it helps to identify and reduced discrepancies between goals and current states (Carver & Scheier, 1998). Although this crucial role of self-awareness’ associated process of goal monitoring for self-regulation has been stressed previously (Harkin et al., 2016), little research has so far investigated how exactly self-awareness impacts goal pursuit via this process of goal monitoring. We bring these lines of research together by referring to literature showing that self-awareness is related to a problem-solving orientation, particularly when goal pursuit becomes difficult (e.g., Kreibich et al., 2021). Hence, self-awareness promotes goal-directed actions by dealing with goal-related difficulties in a problem-solving oriented, thus functional, manner. In line with this, we were able to show that self-awareness protects individuals from experiencing action crises and, as a consequence, contributes to goal-related performance. While a large bunch of literature on self-regulation has so far mainly focused on mechanisms of self-regulation that are common to all individuals (Hoyle, 2006), some literature has pointed out the need to take personality into account when investigating self-regulation due to the fact that self-regulatory processes differ between individuals (Hoyle & Moshontz, 2018). We contribute to this literature and show that (dispositional and situational) variations in self-awareness account for variations in problem-solving and action crises, thus, for a critical part of self-regulation, namely dealing with goal-related difficulties.

### Future directions

Interestingly, some of our analyses showed positive associations between self-awareness and action crisis experience. Specifically, dispositional self-awareness was positively related to action crisis experience in Study 1 and also in Study 2, but only when baseline action crisis was not included as a covariate. Moreover, the positive relations of self-awareness and action crisis were larger than the negative (indirect) effects. In Study 1, the total association of dispositional self-awareness and action crisis was not significant, but the association on the state level was positive. In sum, this suggests that the positive effect dominated in the context of our studies. These results suggest a second mechanism in parallel to problem-solving. As mentioned earlier, there are different conceptualizations of self-awareness (Duval & Wicklund, 1972; Fenigstein et al., 1975; Grant et al., 2002; Trapnell & Campbell, 1999). In our research, we focused on self-awareness as a metacognition (e.g., Grant et al., 2002) with an important role for self-regulation through the associated process of goal monitoring (Carver & Scheier, 1982). However, there is some conceptual overlap and empirical associations with the concept of rumination, a form of maladaptive self-awareness motivated by anxiety and expressed in negative thoughts (Trapnell & Campbell, 1999). Thus, self-aware individuals therefore are more prone to ruminate (e.g., Silvia & Phillips, 2011). As rumination is associated with the experience of action crises (Brandstätter et al., 2013), this fact could explain the positive direct associations between self-awareness and action crisis experience. Future research may investigate whether this mechanism mediates a positive relation between self-awareness and action crises. In this case, the crucial question would be under what circumstances either mechanism prevails, resulting in a net positive or negative impact of self-awareness on action crises. For instance, if there is a lack of instrumental means for problem-solving, the experience of goal-related difficulties might be more likely to promote rumination. If, on the other hand, the means for problem-solving are easily available, problem-solving could be activated. Moreover, while rumination is connected to neuroticism and self-awareness shows associations with openness to experience and conscientiousness (Panah & Seif, 2014), also personality might plays a role.

Moreover, it will be interesting for future research to figure out whether the effects proposed here only go in the direction suggested or also the other way around. Due to the correlational nature of our findings, we cannot make a statement about any causality, that is, whether self-awareness via the process of goal monitoring actually activates problem-solving when goal difficulties arise and whether this process actually reduces the occurrence of action crises. It is well conceivable that experiencing difficulties and action crises in goal pursuit also activates self-awareness. This is because any stimulus that directs attention back on the self can induce a state of self-awareness (Gibbons, 1990). Such self-awareness inducing stimuli could be goal-related stimuli such as difficulties and action crises because personal goals are closely related to the self (Wicklund & Gollwitzer, 1982). In other words, experiencing goal-related difficulties might induce self-awareness which then leads to problem-solving in order to solve these difficulties and prevent action crisis’ experience. Using experimental studies, future lines of research might investigate this idea of such a circular model.

Lastly, one might wonder whether adaptive self-regulation always requires making every effort to ensure that the goal remains attainable. At some point, it might be more adaptive to disengage from the goal (Herrmann & Brandstätter, 2015) and a strong problem-solving orientation may prevent the individual from reconsidering their goal. Earlier research has shown that self-aware individuals are better at deciding whether to invest in a goal pursuit or not, due to their ability to monitor goal progress (Barber et al., 2012). As a consequence, we assume that, if their problem-solving orientation does not result in satisfactory goal progress, self-aware individuals will disengage from the goal rather than experiencing goal-related action crisis. Future research could test these hypotheses empirically.

### Strengths and limitations

This research has several strengths worth highlighting. First, we used a process-oriented approach to investigate individual differences in assessing both individuals’ dispositional and their state levels of self-awareness (Hoyle, 2006). We did so using experience sampling, which is strongly recommended for collecting data on momentary experiences (Mehl & Connor, 2012). Second, we did not rely on self-reporting for all our data but also investigated more objectively assessed outcomes, such as individuals’ exam grades. Third, to increase the generalizability of our findings, we investigated our hypotheses in two very different samples: professional ballet dancers and university undergraduates. Finally, to increase the transparency and reproducibility of our research, we preregistered Study 2 (Mellor & Nosek, 2018) and replicated our findings internally (Asendorpf et al., 2013).

However, except for our performance measurements, all our data relies on self-report scales, some of which are very short. Moreover, the sample size in Study 1 is rather low compared to the sample sizes needed for high stability in correlative effects (Schönbrodt & Perugini, 2018). Furthermore, the demographic of the sample in Study 1 is highly specific. It is important to emphasize that some of the indirect effects are very small, especially the serial indirect effects on goal progress and GPA. This not only limits the practical applicability of our results, but also shows that only a small variance of the experience of an action crisis is explained by the process presented here. Nevertheless, the suggested indirect pathway provides a novel perspective on how self-awareness promotes goal pursuit and performance and thus prevents individuals from experiencing an action crisis. It therefore deepens the understanding on how certain cognitive processes can have a real impact on goal performance. Another limitation is the correlational nature of our findings. Although our longitudinal designs overcomes this limitation to a certain degree, unobserved confounders and alternative causal orders of our concepts of interest cannot be ruled out (see Pek & Hoyle, 2016). Furthermore, it has been shown that a significant mediation analysis result does not prove that the proposed mediator is in fact a mediator (Fiedler et al., 2011). Therefore, the use of experiments to investigate the proposed causal chain is more effective than mediational analyses—at least when the independent variable is easy to manipulate (Spencer et al., 2005). Although previous research provides experimental evidence of a causal effect of self-awareness on problem-solving orientation (Kreibich et al., 2021) and quasi-experimental evidence of the detrimental impact of action crises on performance (Bettschart et al., 2019), future research should therefore investigate the proposed causal chain between self-awareness and action crisis in an experimental setting. Research on self-awareness has provided some first ideas on how to manipulate self-awareness, for instance, by letting participants to write a text about how they differ from others (Silvia & Eichstaedt, 2004) or telling them to monitor their thoughts, feelings, and behavior while doing a certain task (Kreibich et al., 2020).

### Conclusion

This research provides initial evidence that *how* individuals deal with goal-related difficulties directly affect their experience of an action crisis, a phase in goal pursuit that has been shown to have devastating consequences for well-being (e.g., Brandstätter et al., 2013). If individuals deal with such difficulties by searching for and selecting instrumental means instead of, for instance, avoiding the difficulties, the experience of such severe doubts can be reduced. First evidence is furthermore given *who* is able deal like this. It is self-aware individuals who are able to adopt such a problem-solving orientation in goal striving and thus maintain their goals’ attainability and show better goal performance.

## Supplementary Information

Below is the link to the electronic supplementary material.

Supplementary file1 (PDF 74 KB)

## Data Availability

The preregistration of Study 2 can be assessed online at https://aspredicted.org/r33qp.pdf. All study materials are publicly available on the Open Science Framework at https://osf.io/kcx7f/.

## Footnotes

1 The preregistration includes two hypotheses, but only one of them (H1) is the subject of the research reported in this manuscript. Furthermore, we deviate from the preregistration in some minor points because we had better methods available than anticipated. The details can be found in the supplement (see Suppl. Table 3).

2 The primary goal of this study was to test the effect of self-awareness on different dimensions of perfectionism via different mechanisms.

3 We only used items that assess a more general problem-solving orientation and therefore excluded more specific ones that did not fit to the goal we were investigating (e.g., “I do relaxation exercises, meditate or pray.”). Nevertheless, we have reported the results of the analyses using the total scale in the footnotes.

4 Results did not change when using the six-item scale of problem-solving orientation: *b* = 0.25* (a-path), *b* = -0.29* (b-path), *b* = 0.19 (direct effect), *b* = -0.11, 95% CI [-0.30, -0.01] (unstandardized indirect effect).

5 Data and analyses from this study have already been reported in Ghassemi et al. (2020). Except for using the same assessment of action crisis, there is no other overlap with regard to the data or the research questions investigated.

6 The European Credit Transfer System points indicate the approximate workload of a course. One point equals approx. 30 h of work.

7 In the Swiss education system, grades range from 1 to 6. The best possible grade is 6 and grades 4–6 are a pass.

## References

[CR1] Asendorpf, J. B., Conner, M., De Fruyt, F., De Houwer, J., Denissen, J. J. A., Fiedler, K., Fiedler, S., Funder, D. C., Kliegl, R., Nosek, B. A., Perugini, M., Roberts, B. W., Schmitt, M., van Aken, M. A. G., Weber, H., & Wicherts, J. M. (2013). Recommendations for increasing replicability in psychology. *European Journal of Personality*, *27*(2), 108–119. 10.1002/per.1919

[CR2] Barber LK, Grawitch MJ, Munz DC (2012). Disengaging from a task: Lower self-control or adaptive self-regulation?. Journal of Individual Differences.

[CR3] Baumeister RF, Heatherton TF, Tice DM (1994). Losing control: How and why people fail at self-regulation.

[CR4] Bettschart M, Herrmann M, Wolf BM, Brandstätter V (2019). The seed of goal-related doubts: A longitudinal investigation of the roles of failure and expectation of success among police trainee applicants. Frontiers in Psychology.

[CR5] Bishop SR, Lau M, Shapiro S, Carlson L, Anderson ND, Carmody J, Segal ZV, Abbey S, Speca M, Velting D, Devins G (2004). Mindfulness: A proposed operational definition. Clinical Psychology: Science and Practice.

[CR6] Bodenmann G (2000). Stress und Coping bei Paaren [Stress and coping in couples].

[CR7] Bolger N, Laurenceau J-P (2013). Intensive longitudinal methods: An introduction to diary and experience sampling research.

[CR8] Brandstätter V, Schüler J (2013). Action crisis and cost–benefit thinking: A cognitive analysis of a goal-disengagement phase. Journal of Experimental Social Psychology.

[CR9] Brandstätter V, Herrmann M, Schüler J (2013). The struggle of giving up personal goals affective, physiological, and cognitive consequences of an action crisis. Personality and Social Psychology Bulletin.

[CR10] Brown KW, Ryan RM (2003). The benefits of being present: Mindfulness and its role in psychological well-being. Journal of Personality and Social Psychology.

[CR11] Burwell RA, Shirk SR (2007). Subtypes of rumination in adolescence: Associations between brooding, reflection, depressive symptoms, and coping. Journal of Clinical Child & Adolescent Psychology.

[CR12] Carver CS, Scheier MF (1982). Control theory: A useful conceptual framework for personality–social, clinical, and health psychology. Psychological Bulletin.

[CR13] Carver CS, Scheier MF (1998). On the self-regulation of behavior.

[CR14] Curran PJ, Bauer DJ (2010). The disaggregation of within-person and between-person effects in longitudinal models of change. Annual Review of Psychology.

[CR15] Davidson RJ, Dahl CJ (2018). Outstanding challenges in scientific research on mindfulness and meditation. Perspectives on Psychological Science.

[CR16] Duval S, Wicklund RA (1972). A theory of objective self-awareness.

[CR17] Evans DR, Baer RA, Segerstrom SC (2009). The effects of mindfulness and self-consciousness on persistence. Personality and Individual Differences.

[CR18] Fenigstein A, Leary MR, Hoyle RH (2009). Private and public self-consciousness. Handbook of individual differences in social behavior.

[CR19] Fenigstein A, Scheier MF, Buss AH (1975). Public and private self-consciousness: Assessment and theory. Journal of Consulting and Clinical Psychology.

[CR20] Fiedler K, Schott M, Meiser T (2011). What mediation analysis can (not) do. Journal of Experimental Social Psychology.

[CR21] Ghassemi M, Bernecker K, Herrmann M, Brandstätter V (2017). The process of disengagement from personal goals: Reciprocal influences between the experience of action crisis and appraisals of goal desirability and attainability. Personality and Social Psychology Bulletin.

[CR22] Ghassemi M, Wolf BM, Bettschart M, Kreibich A, Herrmann M, Brandstätter V (2020). The dynamics of doubt: Short-term fluctuations and predictors of doubts in personal goal pursuit. Motivation Science, Advance Online Publication..

[CR23] Gibbons FX (1990). Self-attention and behavior: A review and theoretical update. Advances in Experimental Social Psychology.

[CR24] Grant AM, Franklin J, Langford P (2002). The self-reflection and insight scale: A new measure of private self-consciousness. Social Behavior and Personality: An International Journal.

[CR25] Harkin B, Webb TL, Chang BPI, Prestwich A, Conner M, Kellar I, Benn Y, Sheeran P (2016). Does monitoring goal progress promote goal attainment? A meta-analysis of the experimental evidence. Psychological Bulletin.

[CR26] Heckhausen H, Gollwitzer PM (1987). Thought contents and cognitive functioning in motivational versus volitional states of mind. Motivation and Emotion.

[CR27] Herrmann M, Brandstätter V (2013). Overcoming action crises in personal goals—Longitudinal evidence on a mediating mechanism between action orientation and well-being. Journal of Research in Personality.

[CR28] Herrmann M, Brandstätter V (2015). Action crises and goal disengagement: Longitudinal evidence on the predictive validity of a motivational phase in goal striving. Motivation Science.

[CR29] Herrmann M, Baur V, Brandstätter V, Hänggi J, Jäncke L (2014). Being in two minds: The neural basis of experiencing action crises in personal long-term goals. Social Neuroscience.

[CR30] Holding AC, Hope NH, Harvey B, Marion-Jetten AS, Koestner R (2017). Stuck in limbo: Motivational antecedents and consequences of experiencing action crises in personal goal pursuit. Journal of Personality.

[CR31] Hoyle RH (2006). Personality and self-regulation: Trait and information-processing perspectives. Journal of Personality.

[CR32] Hoyle, R. H., & Moshontz, H. (2018, February 8). Self-regulation: An individual difference perspective. 10.31234/osf.io/g25hx

[CR33] Jäkel F, Schreiber C (2013). Introspection in problem solving. The Journal of Problem Solving.

[CR34] Kreibich A, Hennecke M, Brandstätter V (2020). The effect of self-awareness on the identification of goal-related obstacles. European Journal of Personality.

[CR35] Kreibich A, Hennecke M, Brandstätter V (2021). The role of self-awareness and problem-solving orientation for the instrumentality of goal-related means. Journal of Individual Differences.

[CR36] Lazarus RS, Folkman S (1984). Stress, appraisal and coping.

[CR37] Marion-Jetten AS, Taylor G, Schattke K (2021). Mind your goals, mind your emotions: Mechanisms explaining the relation between dispositional mindfulness and action crises. Personality and Social Psychology Bulletin.

[CR38] Martin LL, Tesser A, Wyer RS (1996). Some ruminative thoughts. Ruminative thoughts.

[CR39] Mehl MR, Connor TS (2012). Handbook of research methods for studying daily life.

[CR40] Mellor DT, Nosek BA (2018). Easy preregistration will benefit any research. Nature Human Behaviour.

[CR41] Nolen-Hoeksema S, Wisco BE, Lyubomirsky S (2008). Rethinking rumination. Perspectives on Psychological Science.

[CR42] Oettingen, G., & Gollwitzer, P. M. (2002). Theorien der modernen Zielpsychologie. In D. Frey (Hrsg.), Theorien der Sozialpsychologie (Bd. 3, S. 51–74). Huber.

[CR43] Panah MR, Seif D (2014). Predicting self-awareness dimensions from personality traits among gifted students. Journal of Iranian Psychologists.

[CR44] Pek J, Hoyle RH (2016). On the (in)validity of tests of simple mediation: Threats and solutions. Social and Personality Psychology Compass.

[CR45] R Core Team (2014). R: A language and environment for statistical computing. R Foundation for Statistical Computing, Vienna, Austria. Retrieved from http://www.R-project.org/

[CR46] Schönbrodt FD, Perugini M (2018). Corrigendum to “At what sample size do correlations stabilize?”. Journal of Research in Personality.

[CR47] Silvia PJ, Eichstaedt J (2004). A self-novelty manipulation of self-focused attention for internet and laboratory experiments. Behavior Research Methods, Instruments, & Computers: A Journal of the Psychonomic Society Inc.

[CR48] Silvia PJ, Phillips AG (2011). Evaluating self-reflection and insight as self-conscious traits. Personality and Individual Differences.

[CR49] Simmons, J. P., Nelson, L. D., & Simonsohn, U. (2012, Oktober 14). A 21 word solution. Retrieved from https://ssrn.com/abstract=2160588

[CR50] Spencer SJ, Zanna MP, Fong GT (2005). Establishing a causal chain: Why experiments are often more effective than mediational analyses in examining psychological processes. Journal of Personality and Social Psychology.

[CR51] Tingley D, Yamamoto T, Hirose K, Keele L, Imai K (2014). Mediation: R package for causal mediation analysis. Journal of Statistical Software.

[CR52] Trapnell PD, Campbell JD (1999). Private self-consciousness and the five-factor model of personality: Distinguishing rumination from reflection. Journal of Personality and Social Psychology.

[CR53] Venhorst A, Micklewright DP, Noakes TD (2018). The psychophysiological determinants of pacing behaviour and performance during prolonged endurance exercise: A performance level and competition outcome comparison. Sports Medicine.

[CR54] Wicklund RA, Gollwitzer PM (1982). Symbolic self-completion.

[CR55] Wolf BM, Herrmann M, Brandstätter V (2018). Self-efficacy vs. action orientation: Comparing and contrasting two determinants of goal setting and goal striving. Journal of Research in Personality.

[CR56] Wolf BM, Herrmann M, Zubler I, Brandstätter V (2019). Action crises in personal goals compromise recovery during physical therapy. Motivation Science.

[CR57] Wolf, B. M. (2020). sermed_mlm: R functions to facilitate serial mediation analysis with multilevel data. Retrieved from 10.17605/osf.io/uetn9

