# Supplementary material for: How self-awareness is connected to less experience of action crises in personal goal pursuit
Source: Motiv Emot. 2022 May 23;46(6):825–36. doi: 10.1007/s11031-022-09942-5 (PMC9678988; doi:10.1007/s11031-022-09942-5)
Supplement: Supplementary file 1 — Supplementary file1 (PDF 74 KB) [file 11031_2022_9942_MOESM1_ESM.pdf]

Supplementary Material to

Kreibich, Wolf, Bettschart, Ghassemi, Herrmann, & Brandstätter

How Self-Awareness Connects to Less Action Crises in Personal Goal Pursuit

Methodical details about Study 2

Details on experience sampling phase

The experience sampling phase lasted two weeks excluding weekends, thus 10 days in total. Each day, four of the five brief questionnaires were timed randomly throughout a 12-hour time frame. Participants chose the start of this time frame in the baseline questionnaire (either 7, 8, or 9 a.m.). The fifth brief questionnaire was always sent at the end of this time frame (i.e. at 7, 8, or 9 p.m.).

At the designated times, participants received text messages with a link to the brief questionnaires, which took ca. 2 minutes and had to be answered on a smart phone within 30 minutes before the link expired. Between consecutive messages there was always a time lag of at least 40 min.

We used Survey Signal, an online service for experience sampling studies, to send all text messages accordingly. 2 days prior to the start of the experience sampling phase, participants received an initial text message containing a hyperlink to confirm their mobile number. They had to confirm their mobile until midnight before the start of the experience sampling phase.

Due to a technical failure on the second day of the experience sampling phase, only three out of five messages were sent on that day. Thus reducing the number of brief questionnaires to 48.

Participants had to answer at least 38 (80%) of the brief questionnaires sent during the two weeks. Participants who had a lower response rate (but at least 28 responses) received another 10 brief surveys during the next two workdays. We monitored response rates during the experience sampling phase and contacted participants via text message if they did not answer any brief questionnaires or only very few. In order to save costs, we continuously excluded participants who skipped 19 questionnaires and/or answered that they wanted to quit.

Participants who did not reach the 80% response rate even after extended data collection were excluded from further participation (i.e., the follow-up survey). Participants who reached a 90% response rate overall received a bonus payment (CHF 20) in addition to study

compensation. Participants who were excluded due to technical problems received partial compensation.

Data of participants who voluntarily dropped out or were excluded during the experience sampling phase was used for analysis unless participants asked for their data to be deleted.

Participant dropout and exclusions:

Suppl. Table 1: *Number of participants and exclusions in each phase of Study 2*

Baseline questionnaire (T1)		
Answered T1	263	
Data deleted	1	(Asked for data erasure after exclusion from ES phase)
**Effective N_{T1}**	**262**	
Experience sampling phase		
Excluded from participation	3	1 did not meet participation criteria (no courses to complete)<br>1 spent experience sampling phase abroad<br>1 could not receive messages due to foreign mobile number*
Failed to confirm mobile number	6	
Voluntary dropout before start	1	(Reason unknown)
Started ES phase	253	
**Effective N_{ES}**	**250**	(Answered at least one brief questionnaires)
Voluntary dropout during two weeks	29	1 Lost mobile phone*<br>28 Reasons unknown (missed 19 messages or opted to drop out)
Completed ES phase in two weeks	170	
ES phase extended	54	(Extension by 2 days due to < 80% response rate after 10 days)
Excluded after extended ES phase	9	(Did not reach 38 responses)
Completed ES phase after extension	45	
Bonus payment due to 90% response	70	68 after two-week ES phase<br>2 after extended ES phase
Follow-up questionnaire (T2)		
Invited to and answered T2	215	(Completed ES phase after two weeks or extension)
**Effective N_{T2}**	**215**	

* Received partial compensation of CHF 15
ES: experience sampling

Data usage and exclusion during the experience sampling phase:

Suppl. Table 2: *Number of brief questionnaires submitted, excluded, and used in Study 2*

Brief questionnaires submitted	9280	
Excluded: too fast	31	(among them 4 evening questionnaires)
Not used: evening questionnaires	2016	
Not used: incomplete responses	981	(regarding relevant variables)
Not used: goal-irrelevant activity	2740	
**Effective analysis sample**	**3512**	from N = 250 individual participants

Effective number of brief questionnaires used per participant: M = 14.05, SD = 5.75 (range 1-31)

To ensure that brief questionnaires were not only skimmed through, we excluded responses that were completed within less than 20 seconds (15 seconds for evening

questionnaires). We also intended to exclude any responses that took more than 30 minutes but none did.

Among the five brief questionnaires sent out every day, the fifth one (evening questionnaire) did not contain all relevant variables and is therefore not used for the present analyses. Unfortunately, while monitoring the data collection, we could not distinguish between complete and partially answered questionnaires. Therefore, a relevant proportion of our data has missing values for one or several of the relevant variables and could not be used. Furthermore, we were only interested in moments when participants were actually engaged in an activity related to their academic goal.

Deviations from preregistered analysis procedure in Study 2:

Suppl. Table 3: *Preregistered and reported analysis methods in Study 2*

Hypothesis	***Preregistered method***	***Reported method***	***Rationale***
Baseline (& follow-up) data:<br>Baseline problem solving orientation mediates the negative association between self-awareness at the baseline and action crisis in the follow-up survey.	Mediation analysis using PROCESS macro for SPSS by Hayes (2001).	Mediation analysis using “mediation” package for R (Tingley et al., 2014).	Using consistent software for all analyses.
Experience sampling data:<br>Momentary problem solving orientation mediates the negative association between momentary self-awareness and doubts.	Clustered mediation analysis using PROCESS macro for SPSS by Hayes (2012).<br><br>Analysis of data on the level of *days*: Experience sampling and problem solving each aggregated across up to 4 questionnaires per day; progress measured in the 5th (evening) questionnaire.	Multilevel serial mediation analysis using sermedMLM by the second author in R (available on OSF: [link omitted for blind review]).<br><br>Used data on the level of *brief questionnaires* (up to 4 times per day).	Using sermedLM allowed us (1.) to disentangle within- and between person effects and (2.) to extend the mediation model and include progress as a subsequent dependent variable.<br><br>Brief questionnaire data allowed more fine-grained analysis than aggregated data per day. Furthermore, daily aggregates may include data from varying numbers of brief questionnaires, thus essentially weighting individual responses differentily.

Note: The preregistration for Study 2 is available under https://aspredicted.org/blind.php?x=ci3dm8

Descriptive Statistics on Study 2:

Suppl. Table 4: *Means, standard deviations, and correlations of person-level variables*

Variable	*M*	*SD*	1	2	3	4	5	6	7	8	9
1. Trait self-awareness	4.57	0.77									
2. State self-awareness [a]	3.10	1.18	.37**								
3. Dispositional PSO	3.66	0.69	.20**	.02							
4. State PSO [a]	3.98	0.97	.12	.32**	.08						
5. Action crisis baseline	2.99	1.06	.07	.20**	-.21**	-.08					
6. Action crisis follow-up	2.92	0.96	.08	.23**	-.26**	.01	.71**				
7. Doubt [a]	1.82	1.05	.06	.25**	-.21**	-.03	.64**	.74**			
8. Goal progress [a]	4.80	0.71	.12	.05	.11	.41**	-.21**	-.22**	-.26**		
9. Semester GPA	4.72	0.88	-.05	-.07	.01	-.03	-.29**	-.32**	-.23**	.09	
10. Semester ECTS	22.77	8.42	-.07	-.03	.09	.07	-.18*	-.11	-.06	.04	.11

Note: $N = 215$, except for GPA ($N = 189$) and ECTS ($N = 215$)
PSO: Problem-solving orientation
[a] Person mean throughout the experience sampling phase
* indicates $p < .05$. ** indicates $p < .01$.

Suppl. Table 5: *Means, standard deviations, and correlations of experience-sampling phase variables*

Variable	*M*	*SD*	1	2	3
1. State self-awareness	3.07	1.69			
2. State PSO	3.98	1.67	.18**		
3. Doubt	1.78	1.06	.12**	-.02	
4. Goal progress	4.65	1.43	.00	.30**	-.19**

Note: $N = 3512$ brief surveys from 250 participants
PSO: Problem-solving orientation
* indicates $p < .05$. ** indicates $p < .01$.

Extended Results from Study 2:

Suppl. Table 6: *Regression models of the between-persons mediation analyses in Study 2 with (A) and without (B) baseline action crisis as a covariate*

Version A: with covariate (reported in Fig. 2)

Dependent: Problem-solving orientation (T1)				
	Coef.	*SE*	*t*	*p*
(intercept)	2.83	0.28	10.184	< .001
Self-awareness (T1) – *a* path	0.18	0.06	3.033	.003
Dependent: Action crisis (T2)				
	Coef.	*SE*	*t*	*p*
(intercept)	1.42	0.37	3.845	< .001
Self-awareness (T1) – *c‘* path	0.07	0.06	1.155	.249
Problem-solving orientation (T1) – *b* path	-0.18	0.07	-2.622	.009
Action crisis (T1)	0.62	0.04	13.976	< .001
		95% CI		
Indirect effect – *a*b*	-0.03	[-0.07, -0.01]		

Version B: without covariate

Dependent: Problem-solving orientation (T1)				
	Coef.	*SE*	*t*	*p*
(intercept)	2.83	0.28	10.184	< .001
Self-awareness (T1) – *a* path	0.18	0.06	3.033	.003
Dependent: Action crisis (T2)				
	Coef.	*SE*	*t*	*p*
(intercept)	3.62	0.45	7.830	< .001
Self-awareness (T1) – *c‘* path	0.17	0.08	2.025	.044
Problem-solving orientation (T1) – *b* path	-0.40	0.09	-4.315	< .001
		95% CI		
Indirect effect – *a*b*	-0.07	[-0.14, -0.02]		

Note:
T1 – baseline survey; T2 – follow-up survey;
Coef. – regression coefficient estimate; *SE* – standard error of regression coefficient

Suppl. Table 7: *Multilevel regression models of the serial mediation analysis with experience sampling data in Study 2*

Dependent: Momentary problem-solving orientation				95% CI_{boot}	
	Coef.	*SE*	*t*	*lower*	*upper*
(intercept)	3.94	0.06			
within persons:			(df = 3261)		
Self-awareness – $a_{1with.}$ path	0.11	0.02	5.511	0.065	0.148
between persons:			(df = 248)		
Self-awareness – $a_{1betw.}$ path	0.27	0.05	5.445	0.231	0.315
Random effects:	*SD*				
Intercept	0.836		log Lik.	AIC	BIC
Residual	1.414		-6418.7	12847	12878
**Dependent: Doubts**				95% CI_{boot}	
	Coef.	*SE*	*t*	*lower*	*upper*
(intercept)	1.77	0.06			
within persons:			(df = 3260)		
Self-awareness	0.02	0.01	3.334	0.007	0.033
Problem-solving orientation – $a_{2with.}$ path	-0.02	0.01	-3.209	-0.027	-0.007
between persons:			(df = 247)		
Self-awareness	0.23	0.06	4.145	0.208	0.249
Problem-solving orientation – $a_{2betw.}$ path	-0.09	0.07	-1.412	-0.110	-0.077
Random effects:	*SD*				
Intercept	0.972		log Lik.	AIC	BIC
Residual	0.430		-2556.5	5127	5170
**Dependent: Subjective progress**				95% CI_{boot}	
	Coef.	*SE*	*t*	*lower*	*upper*
(intercept)	4.68	0.04			
within persons:			(df = 3259)		
Self-awareness	-0.03	0.02	-2.041	-0.070	0.002
Problem-solving orientation	0.24	0.02	16.04	0.204	0.271
Problem-solving orientation – $b_{with.}$ path	-0.31	0.05	-6.269	-0.407	-0.192
between persons:			(df = 246)		
Self-awareness	-0.03	0.04	-0.747	-0.074	0.009
Problem-solving orientation	0.30	0.05	6.497	0.253	0.355
Problem-solving orientation – $b_{betw.}$ path	-0.20	0.04	-4.436	-0.238	-0.155
Random effects:	*SD*				
Intercept	0.588		log Lik.	AIC	BIC
Residual	1.198		-5809.6	11637	11693

Note: T1 – baseline survey; T2 – follow-up survey;
Coef. – regression coefficient estimate; *SE* – standard error of regression coefficient;
CI_{boot} – bias-corrected bootstrap confidence interval (5000 samples);
SD – standard deviation of random effects; log Lik. – log of likelihood;
AIC – Akaike information criterion; BIC – Bayesian information criterion
The indirect effects on doubts are the products of the a_1 and a_2 paths:
$a_{1with.}a_{2with.}$ = -0.002, 95% CI_{boot}: [-0.003, -0.001]
$a_{1betw.}a_{2betw.}$ = -0.026, 95% CI_{boot}: [-0.032, -0.020]
The serial indirect effects on progress are the products of the a_1, a_2, and b paths:
$a_{1with.}a_{2with.}b_{with.}$ = 0.0006, 95% CI_{boot}: [0.0002, 0.0012]
$a_{1betw.}a_{2betw.}b_{betw.}$ = 0.005, 95% CI_{boot}: [0.004, 0.007]

Suppl. Table 8: *Regression models of the between-persons serial mediation analysis in Study 2*

Dependent: Problem-solving orientation (T1)					95% CI_{boot}	
	Coef.	*SE*	*t*	*p*	*lower*	*upper*
(intercept)	2.83	0.31	9.265	< .001		
Self-awareness (T1) – a_1 path	0.18	0.07	2.780	.006	0.035	0.341
**Dependent: Action crisis (T2)**					95% CI_{boot}	
	Coef.	*SE*	*t*	*p*	*lower*	*upper*
(intercept)	3.52	0.50	7.023	< .001		
Self-awareness (T1)	0.16	0.09	1.793	.075	-0.023	0.354
Problem-solving orientation (T1) – a_2 path	-0.38	0.10	-3.791	< .001	-0.614	-0.168
**Dependent: Semester GPA**					95% CI_{boot}	
	Coef.	*SE*	*t*	*p*	*lower*	*upper*
(intercept)	6.02	0.51	11.787	< .001		
Self-awareness (T1)	-0.01	0.08	-0.104	.917	-0.157	0.135
Problem-solving orientation (T1)	-0.10	0.09	-1.026	.306	-0.292	0.085
Action crisis (T2) – *b* path	-0.32	0.07	-4.747	< .001	-0.483	-0.181

Note:
T1 – baseline survey; T2 – follow-up survey;
Coef. – regression coefficient estimate; *SE* – standard error of regression coefficient;
CI_{boot} – bias-corrected bootstrap confidence interval (5000 samples)
The indirect effect on action crisis (T2) is the product of the a_1 and a_2 paths:
a_1a_2 = -0.069, 95% Ci_{boot}: [-0.171, -0.014]
The serial indirect effect on semester GPA is the product of the a_1, a_2, and *b* paths:
a_1a_2b = 0.022, 95% CI_{boot}: [0.004, 0.060]

Suppl. Table 9: *Regression models of the between-persons serial mediation analysis in Study 2 including baseline action crisis as control*

Dependent: Problem-solving orientation (T1)					95% CI_{boot}	
	Coef.	*SE*	*t*	*p*	*lower*	*upper*
(intercept)	2.83	0.31	9.265			
Self-awareness (T1) – a_1 path	0.18	0.07	2.780	0.006	0.041	0.346
**Dependent: Action crisis (T2)**					95% CI_{boot}	
	Coef.	*SE*	*t*	*p*	*lower*	*upper*
(intercept)	1.13	0.36	3.102			
Self-awareness (T1)	0.02	0.06	0.317	0.752	-0.102	0.134
Problem-solving orientation (T1) – a_2 path	-0.10	0.07	-1.481	0.140	-0.260	0.054
Action crisis (T1)	0.70	0.04	15.691	< .001		
**Dependent: Semester GPA**					95% CI_{boot}	
	Coef.	*SE*	*t*	*p*	*lower*	*upper*
(intercept)	6.07	0.51	11.838			
Self-awareness (T1)	0.00	0.08	-0.017	0.987	-0.143	0.142
Problem-solving orientation (T1)	-0.10	0.09	-1.116	0.266	-0.299	0.076
Action crisis (T1)	-0.10	0.09	-1.063	0.289		
Action crisis (T2) – *b* path	-0.23	0.10	-2.307	0.022	-0.471	-0.066

Note:
T1 – baseline survey; T2 – follow-up survey;
Coef. – regression coefficient estimate; *SE* – standard error of regression coefficient;
CI_{boot} – bias-corrected bootstrap confidence interval (5000 samples)
The indirect effect on action crisis (T2) is the product of the a_1 and a_2 paths:
a_1a_2 = -0.018, 95% CI_{boot}: [-0.069, 0.005]
The serial indirect effect on semester GPA is the product of the a_1, a_2, and *b* paths:
a_1a_2b = -0.004, 95% CI_{boot}: [-0.001, 0.021]

References

Hayes, A. F. (2012). *PROCESS: A versatile computational tool for observed variable mediation, moderation, and conditional process modeling* [white paper]. http://www.afhayes.com/public/process2012.pdf

Tingley, D., Yamamoto, T., Hirose, K., Keele, L., & Imai, K. (2014). mediation: R Package for Causal Mediation Analysis. *Journal of Statistical Software*, *59*(1), 1–38. https://doi.org/10.18637/jss.v059.i05
